# Co-Occurrence of Pheochromocytoma-Paraganglioma and Cyanotic Congenital Heart Disease: A Case Report and Literature Review

**DOI:** 10.3389/fendo.2018.00165

**Published:** 2018-04-17

**Authors:** Bingbin Zhao, Yi Zhou, Yi Zhao, Yumo Zhao, Xingcheng Wu, Yalan Bi, Yufeng Luo, Zhigang Ji, Shi Rong

**Affiliations:** ^1^Peking Union Medical College Hospital, Peking Union Medical College, Chinese Academy of Medical Sciences, Beijing, China; ^2^Department of Urology, Peking Union Medical College Hospital, Peking Union Medical College, Chinese Academy of Medical Sciences, Beijing, China; ^3^Department of Pathology, Peking Union Medical College Hospital, Peking Union Medical College, Chinese Academy of Medical Sciences, Beijing, China

**Keywords:** pheochromocytoma, paraganglioma, cyanotic congenital heart disease, hypoxia, hypoxia-inducible factor

## Abstract

Pheochromocytoma and paraganglioma (PHEO-PGL) and cyanotic congenital heart disease (CCHD) are both rare diseases. We reported a 30-year-old patient with a right adrenal gland nodule and a retroperitoneal mass and history of functional single atrium and ventricle. ^123^I-metaiodobenzylguanidine scintigraphy showed intense uptake in both lesions. Laboratory investigation demonstrated elevated urinary norepinephrine. Preoperative α-blockade was initiated. A successful open resection of right adrenal and retroperitoneal masses was performed. Pathological examination confirmed PHEO-PGL. Postoperative urinary norepinephrine returned to normal level. A systematic case review in English publications in PubMed and EMBASE suggested a hypothesis that there may exist a possible link between PHEO-PGL and hypoxia from CCHD, which was also indicated in our case. Due to higher risk for PHEO-PGL, a lower threshold of suspicion should be considered in CCHD patients. Therefore, active screening and early treatment of PHEO-PGL are recommended in CCHD patients and clinicians should keep on a long-term follow-up to monitor PHEO-PGL recurrence if hypoxia is not corrected.

## Introduction

Pheochromocytoma and paraganglioma (PHEO-PGL) are rare neuroendocrine tumors derived from either chromaffin cells in adrenal medulla and extra-adrenal sympathetic ganglia or non-chromaffin cells in parasympathetic tissue, including the carotid body. PHEO and PGL arising from the sympathetic chain commonly produce catecholamines in contrast to those from parasympathetic tissues (1). It was estimated that the prevalence of PHEO-PGL in patients with hypertension ranged from 0.2 to 0.6% (2).

Cyanotic congenital heart disease (CCHD) refers to a group of heart defects presenting at birth, which causes low level of oxygen in blood and leads to chronic hypoxemia. CCHD may be responsible for the multisystem disorders, including secondary erythrocytosis (3). The reported incidence of CCHD in live births was 0.12% (4).

Here, we presented a unique case of a patient who was diagnosed with PHEO-PGL and had a history of CCHD. Additionally, a literature review of similar reported cases was performed and is discussed.

## Case Report

A 30-year-old woman was presented to the urology outpatient clinic for assessment after an incidental finding of a retroperitoneal mass and a nodule in the right adrenal gland.

**Figure 1:**
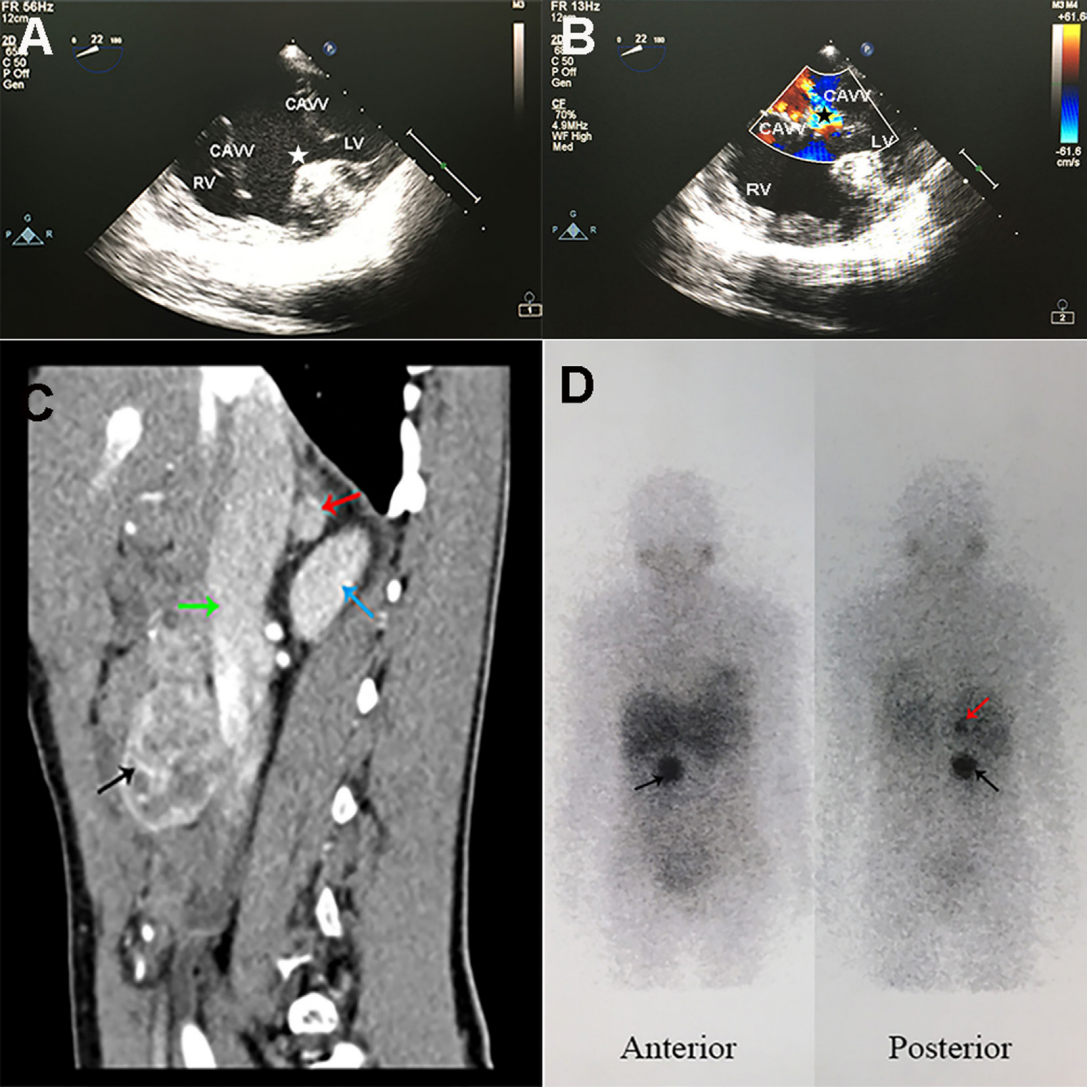
Specific radiographic imaging of the patient. **(A,B)** Trans-esophageal echocardiography showed functional single ventricle and severe common atrioventricular valve regurgitation. **(A)** CAVVs were open in cardiac diastole, and huge ventricular septal defect (white star) was observed. **(B)** CAVVs were closed in cardiac systole and regurgitation of blood into the ventricle (black star) was observed. RV, right ventricle; LV, left ventricle; CAVV, common atrioventricular valve. **(C)** Contrast-enhanced computed tomography sagittal reconstruction image of the abdomen showed a rounded mass (1.1 cm × 1.1 cm × 1.1 cm) in right adrenal gland (red arrow) and another mass (4.4 cm × 3.2 cm × 4.3 cm) with mixed density (black arrow) anterior to inferior vena cava (green arrow) at L2–3 lumbar level. The right kidney was showed with blue arrow. **(D)** ^123^I-MIBG scintigraphy showed intense uptake in the right adrenal gland (red arrow) and in front of the inferior vena cava (black arrow) at L2–3 lumbar level.

She was diagnosed with cyanotic complex congenital heart disease at birth. At age 13, her arterial saturation was measured 74.6% (PO_2_ 46.6 mmHg) with a hemoglobin (HGB) of 197 g/L. At age 25, bilateral bidirectional Glenn (cavopulmonary shunt) was performed, after which cyanosis was alleviated with improved exercise capacity evaluated as class II by New York Heart Association (NYHA) classification (5). Recent echocardiography (Figures 1A,B) showed double outlet right ventricle, complete atrioventricular canal defect, severe common atrioventricular valve regurgitation, and pulmonary valve stenosis, indicating a functional single atrium and ventricle. At age 28, she had a cesarean section delivery of a 4.8 kg baby at full term. Her baby was otherwise healthy. Her family history was unremarkable.

On admission, her blood pressure (BP) was 102/76 mmHg and pulse oximetry indicated that her blood oxygen saturation was 76%. She presented with cyanotic lips and clubbed fingers. A III/VI systolic murmur was detected in the precordial area. There were no other positive findings on physical examination.

Arterial blood gas analysis showed an oxygen saturation of 85.2% (PO_2_ 49.4 mmHg). Pertinent urinary laboratory findings included the followings: elevated norepinephrine level (123.75 µg/day; normal range, 16.69–40.65), normal epinephrine (3.38 µg/day), and dopamine (208.42 µg/day) levels. Her HGB was 106 g/L (110–150) and the erythropoietin (EPO) was markedly elevated to 250.99 mIU/ml (4.5–31.88). The plasma creatinine and urea were within the normal range. A computed tomography (CT) scan of the abdomen and pelvis (Figure 1C) demonstrated a rounded mass (1.1 cm × 1.1 cm × 1.1 cm) in the right adrenal gland and another mass (4.4 cm × 3.2 cm × 4.3 cm) with mixed density anterior to inferior vena cava at L2–3 lumbar level. The contrast-enhanced CT scan showed remarkable heterogeneous enhancement in the arterial phase. Marked accumulation of ^123^I-metaiodobenzylguanidine (MIBG) (Figure 1D) in the right adrenal gland and in front of the inferior vena cava was found. There were no positive findings in ^99m^Tc-HYNIC-TOC single photon emission computed tomography (SPECT)/CT used for detecting somatostatin receptor expression. A clinical diagnosis of pheochromocytoma in the right adrenal gland and a retroperitoneal paraganglioma was made, with no metastases found at other non-chromaffin sites.

Preoperative management was initiated with oral administration of phenoxybenzamine 5 mg twice daily for 3 weeks. Open resection of retroperitoneal paraganglioma and pheochromocytoma of right adrenal gland was performed. BP, pulse rate, and cardiac function monitored by trans-esophageal echocardiography were stable during the operation. Postoperatively, urinary norepinephrine was corrected (29.08 μg/day).

The resected right adrenal gland nodule was 1.2 cm in diameter (Figures 2A,B). The retroperitoneal mass was 4.6 cm × 3.3 cm × 4.5 cm (Figures 2A,B) with multifocal areas of necrosis on the cut surface. Microscopically, the tumor cells (Figures 2C,D) had slightly basophilic cytoplasm. The immunohistochemistry test was positive for chromogranin (Figures 2E,F), S-100 (Figures 2G,H), and HIF2α (Figures 2I,J). The diagnosis of PHEO-PGL was confirmed. No germline mutation was detected in *NF1, VHL, RET, SDHA, SDHB, SDHC, SDHD, SDHAF2, MAX, TMEM127, FH, KIF1B, BAP1, HIF2A, EGLN1, EGLN2, EGLN3, HRAS, IDH1*, and *MDH2* using targeted next-generation sequencing (Illumina NextSeq 500 platform, Illumina, San Diego, CA, USA).

**Figure 2:**
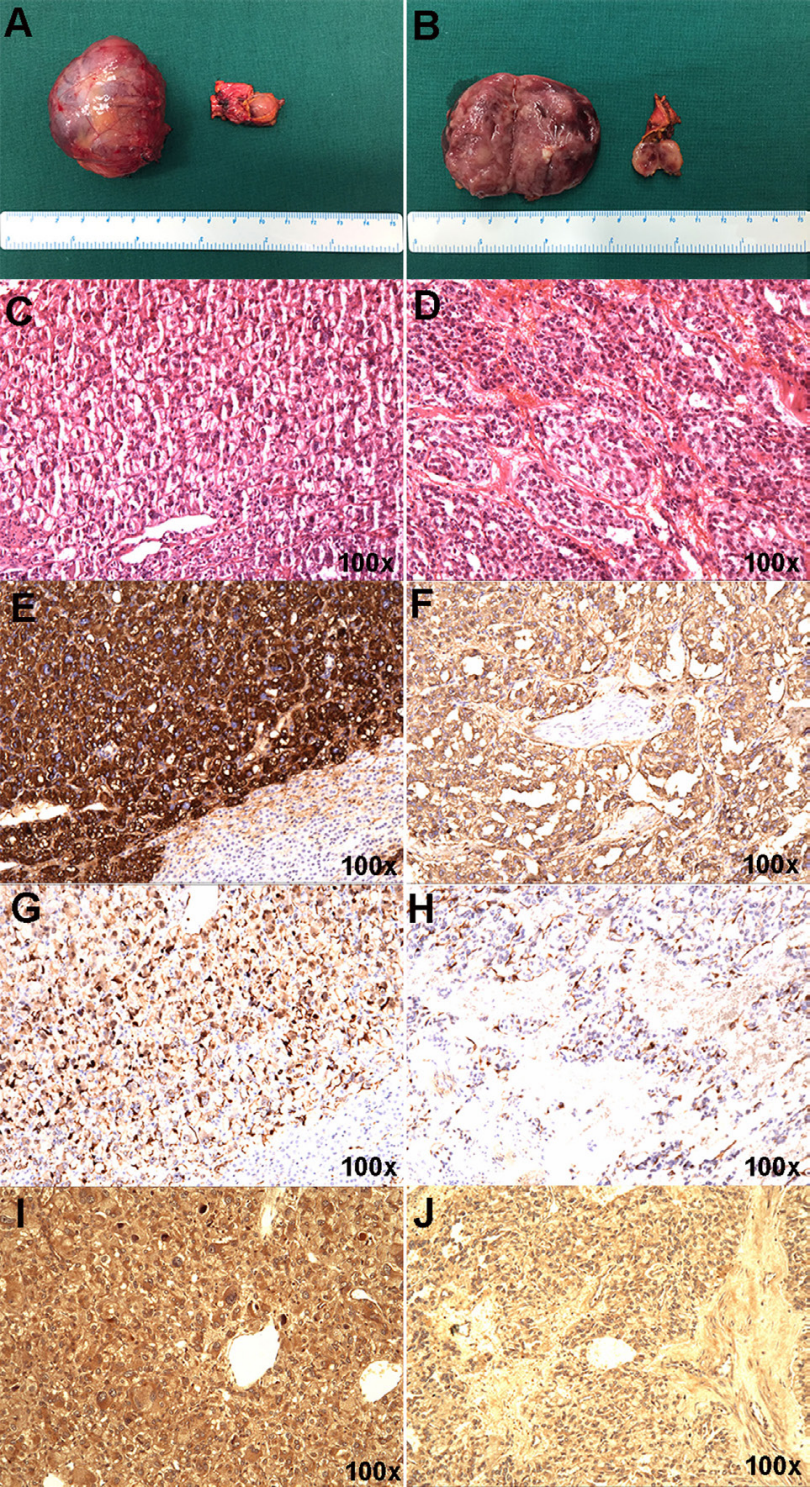
Gross appearance and histopathology of the resected pheochromocytoma and paraganglioma masses. **(A,B)** The right adrenal gland nodule was about 1.2 cm in diameter, connecting to part of normal adrenal gland tissue. Hematoxylin and eosin staining **(C)**, chromogranin **(E)**, S-100 **(G)**, and HIF-2α **(I)** in the right adrenal gland nodule. **(A,B)** The retroperitoneal mass was about 4.6 cm × 3.3 cm × 4.5 cm in size and the section showed partial necrosis. Hematoxylin and eosin staining **(D)**, chromogranin **(F)**, S-100 **(H)**, and HIF2α **(J)** in the retroperitoneal mass.

Our case was carried out in accordance with the recommendations of the Ethics Committee on Human Studies at Peking Union Medical College Hospital (PUMCH). The written informed consent was obtained from the patient.

## Case Review

For the literature review, we searched PubMed and EMBASE for case reports of patients with both PHEO-PGL and CCHD using the term: *pheochromocytoma, paraganglioma, congenital heart disease, and cyanotic congenital heart disease*. We identified 47 such patients with full-text access up to November 20, 2017. Data were expressed as mean ± SD or percentages, unless stated otherwise. Differences were analyzed using *t*-test. Significance level was set at 0.05. All statistical analyses were performed with SPSS 17.0 statistical software (SPSS, Chicago, IL, USA).

Characteristics of these patients were listed in Table 1 and Table S1 in Supplementary Material. Mean age at diagnosis of PHEO-PGL in these patients was 29.5 ± 13.6 years, younger than the mean age (40 years) of those without other diseases (1), and the interval between the onset of cyanosis and PHEO-PGL was 25.5 ± 10.4 years. Among the patients, 12.8 and 10.6% had multiple lesions and metastases, respectively. Mean oxygen saturation was 77.6 ± 12.0%. These characteristics were consistent with findings from a multicenter case series with 18 patients (6). According to whether the cyanosis resulting from congenital heart defects appeared immediately after birth (7), we classified the patients into two groups: patients who had cyanosis at birth and those who had Eisenmenger’s syndrome. Further analysis showed that patients undergoing hypoxia from birth were diagnosed with PHEO-PGL at a younger age than those in the other group (26.6 ± 11.7 vs 46.3 ± 12.2 years, *P* = 0.004). Moreover, the percentage of secretive tumors (PHEO and PGL from sympathetic ganglia) was higher among patients with cyanosis at birth compared to later developed group (*P* = 0.001). Clinical genetic testing results were available for only three cases and no germline mutation was detected.

**Table 1:** Summary characteristics of pheochromocytoma and paraganglioma (PHEO-PGL) patients with cyanotic congenital heart disease.

	Cyanosis at birth	Eisenmenger’s syndrome	Overall
*n*	40	7	47
Sex (male: female)	16:24	0:7	16:31
Age at diagnosis of PHEO-PGL (years)	26.6 (11.7)	46.3 (12.2)	29.5 (13.6)
Oxygen saturation (%)	76.8 (12.5)	83.8 (4.8)	77.6 (12.0)^a^
Hypoxic duration (years)	25.6 (10.6)	24.0 (11.3)	25.5 (10.4)^b^
Types of PHEO-PGL			
PHEO	21 (52.5%)	1 (14.3%)	22 (46.8%)
PGL	18 (47.5%)	6 (85.7%)	24 (51.1%)
PHEO and PGL	1 (2.5%)	0 (0)	1 (2.1%)
Numbers of lesions			
Single without metastases	30 (75%)	6 (85.7%)	36 (76.6%)
Multiple without metastases	5 (12.5%)	1 (14.3%)	6 (12.8%)
With metastases	5 (12.5%)	0 (0)	5 (10.6%)

*Data are *n* (%) or mean (SD), unless stated otherwise. All statistical analyses were performed with SPSS 17.0 statistical software (SPSS, Chicago, IL, USA)*.

*^a^n = 35, arterial oxygen saturation levels of the other 12 patients were not described in the cases*.

*^b^n = 42, hypoxic years of the other five patients were not described in the cases*.

## Discussion

Co-occurrence of CCHD and PHEO-PGL has been repeatedly reported, since 1964 (8), but whether there exists potential association between CCHD and PHEO-PGL remains controversial. A population-based cross-sectional analysis provided direct evidence for the relevance that patients with CCHD had a higher risk of developing PHEO-PGL (odds ratio: 6.0) than those with noncyanotic congenital heart disease (odds ratio: 0.9), each compared with patients without CHD (6). Our case review also demonstrated that CCHD patients were diagnosed with PHEO-PGL at a younger age than overall population. The underlying mechanism may be the hypoxia resulted from CCHD. In 1973, Saldana et al. (9) found that the prevalence of PGL in high altitude areas was 10 times higher than at sea level areas. Gruber et al. (10) reported a case with bilateral carotid body PGLs and chronic hypoxemia, and the tumor regressed after improvement of oxygenation. It is reasonable to speculate that PGL could be the result of reactive hyperplastic growth for adaptation to hypoxia (11–13). However, it is early to assume that oxygenation improvement can alleviate PHEO-PGL due to lack of evidence.

It was reported that up to 70–80% patients with PHEO-PGL had a genetic driver event, considering germline and somatic mutations together (14, 15). According to the Cancer Genome Atlas (TCGA) molecular taxonomy (16), the known genes were divided into three main clusters: pseudo-hypoxia group, including *SDHA, SDHB, SDHC, SDHD, SDHAF2, VHL*, and *HIF2*α; Wnt signaling group, including *CSDE1* and *MAML3*; kinase signaling group, including *RET, NF1, TMEM127, MAX*, and *HRAS*. The pseudo-hypoxic group was associated with dysregulation of hypoxia-inducible factor (HIF), an oxygen-liable transcription factor in the pathway of cellular responses to hypoxia, which could modulate a wide variety of target genes in the regulation of angiogenesis, apoptosis, proliferation, energy metabolism, and migration (17–20). In our case, general HIF2α positivity observed in the resected tissues may be attributed to the chronic hypoxia of the patient (19, 21–23). This provided possible molecular evidence for the relevance between the hypoxic pathway and development of PHEO-PGL (24). Eisenhofer et al. (25) assessed catecholamine biochemical phenotypes in different PHEO-PGL and found patients with *VHL* and *SDH* mutations (pseudo-hypoxia group) were characterized by noradrenergic biochemical phenotype, while patients with *NF1* and *RET* mutations usually showed adrenergic phenotype. Our patient presented with a noradrenergic phenotype, indicating probable similarity between pseudo-hypoxia and chronic hypoxia in CCHD. Another hypothesis suggested that the co-occurrence resulted from the disrupted development and differentiation of neural crest cells, the common origin of adrenal medulla and outlet tract of the heart (26). Interestingly, HIF-2α had been suggested to play an important role in the development of the adrenal medulla and the paraganglia (27). Overall, despite the fact that the mechanism was not fully clarified yet, HIFs may play a central role in the tumorigenesis of PHEO-PGL.

Clinical presentations of PHEO-PGL are variable, and classic manifestations, include episodes of palpitation, headache, and profuse sweating, which are greatly related to catecholamine release (2). Additionally, as our patient presented, PHEO-PGL can be asymptomatic for years and found incidentally. It was reported that catecholamine overproduction can be observed in 66% of the asymptomatic patients (28, 29). Catecholamine crises can lead to cardiovascular complications, including heart failure, progressive arrhythmia which can deteriorate cardiovascular conditions in CCHD patients, and even to sudden cardiac death (1, 6). Concerning the management of patients with PHEO-PGL and CCHD, multi-disciplinary team, including endocrinology, urology, cardiology, anesthesiology, and ICU is critical to allow successful surgical resection and achieve remission.

Better understanding of the pathophysiology of PHEO-PGL has led to clinical suggestions. First, CCHD patients are at higher risk of development of PHEO-PGL due to chronic hypoxic exposure. Therefore, active screening and early treatment for PHEO-PGL by biochemical or radiological methods may be beneficial for CCHD patients. Second, the therapeutic strategies targeting HIFs pathway can be promising for PHEO-PGLs, especially in refractory cases (30). Third, long-term follow-up of recurrence after resection may provide evidence of the association between CCHD and PHEO-PGL. Also, whole-exome sequencing may further uncover the underlying mechanisms of the co-occurrence of PHEO-PGL and CCHD.

## Conclusion

Multidisciplinary cooperation is critical for the management of patients with PHEO-PGL and CCHD, and long-term follow-up after surgery maybe a necessity to monitor the recurrence. Further research is needed for better understanding and revealing the deeper pathogenic connection between hypoxia and PHEO-PGL.

## Ethics Statement

All procedures and medications of the patients were based on clinical guidelines and out of the interests of patients. And according to the recommendations of the Ethics Committee on human studies at Peking Union Medical College Hospital (PUMCH), it can be exempt from ethical approval procedures. Also, the patient gave written informed consent on the publication of the case and also signed the informed consent made by clinical specimen administration center of PUMCH on the use of specimen resected during the operation and the serial number of the informed consent was PUMCHBC-C-4-Q02-1.

## Author Contributions

BZ and YZ (Zhou) are the co-first authors for this case report. SR is the corresponding author supervising this work. SR, BZ, YZ (Zhou), and XW managed the case. BZ, YMZ, and YZ (Zhao) performed analysis on all data interpretation from literature review and drafted the manuscript. SR, ZJ, YZ (Zhou), XW, and YZ (Zhao) reviewed the manuscript. YB and YL prepared histopathological results.

## Conflict of Interest Statement

The authors declare that the research was conducted in the absence of any commercial or financial relationships that could be construed as a potential conflict of interest.
