# Supplementary material for: Co-Occurrence of Pheochromocytoma-Paraganglioma and Cyanotic Congenital Heart Disease: A Case Report and Literature Review
Source: Front Endocrinol (Lausanne). 2018 Apr 17;9:165. doi: 10.3389/fendo.2018.00165 (PMC5914282; doi:10.3389/fendo.2018.00165)
Supplement: Supplementary file 1 [file table_1.DOCX]

***Supplementary Material***

**Pheochromocytoma and Paraganglioma Complicated with Cyanotic Congenital Heart Disease: a case report and literature review**

Bingbin Zhao, Yi Zhou, Yi Zhao, Yumo Zhao, Xingcheng Wu, Yalan Bi, Yufeng Luo, Zhigang Ji, Shi Rong*

***Correspondence:** Shi Rong: rongspumch@126.com

**Supplemental Table 1.** Case review: PHEO-PGL patients with CCHD. Abbreviations: SV, single ventricle; TA, tricuspid atresia; PS, pulmonary stenosis; SVC, superior vena cava; DORV, double-outlet right ventricle; VSD, ventricular septal defect; TGA, transposition of the great arteries; IVC, inferior vena cava; SA, single atrium; PA, pulmonary atresia; ASD, atrial septal defect; TOF, tetralogy of Fallot; ECD, endocardial cushion defect; PAS, pulmonary artery stenosis; PAH, pulmonary arterial hypertension; PDA, patent ductus arteriosus; ND, no data; NE, norepinephrine; E, epinephrine; DA, dopamine; PHEO, pheochromocytoma; PGL, paraganglioma.

| **Case** | **Sex** | | **Age at diagnosis** | **Cyanotic heart disease** | **SO_2_, %** | **PaO_2_, mmHg** | **Hypoxic years** | **Elevated catecholamine** | **Tumor** | **Ref.** |
| --- | --- | --- | --- | --- | --- | --- | --- | --- | --- | --- |
| 1 | M | | 58 | common ventricle with high pulmonary vascular resistance | ND | 43 | 58 | ND | Multiple PGL | 1 |
| 2 | M | | 13 | SV, TA, PS, dysplasic single atrioventricular valve and duplication of SVC | 83 | ND | 13 | NE | Multiple PGL | 2 |
| 3 | M | | 25 | DORV, VSD, PS | 82 | ND | 25 | NE/E | Multiple PGL | 3 |
| 4 | F | | 12 | TGA, VSD | 30-50 | ND | 12 | NE | Multiple PGL with metastases | 4 |
| 5 | F | | 20 | visceral situsinversus with isolated levocardia, absence of IVC, SV, SA, PA and aortopulmonary collaterals | 80 | ND | 20 | NE | Multiple PHEO | 5 |
| 6 | F | | 43 | situsinversus, dextrocardia, VSD, ASD, PA, overriding aorta | 75 | ND | 43 | NE | PHEO, PGL | 6 |
| 7 | M | | 26 | TOF | 54 | ND | 26 | ND | Single PGL | 7 |
| 8 | M | | 20 | TOF | 80 | ND | 20 | NE | Single PGL | 7 |
| 9 | M | | 52 | SV, PS | ND | ND | 52 | ND | Single PGL | 8 |
| 10 | F | | 16 | TOF, complete ECD, SA | 76.7 | ND | 16 | NE | Single PGL | 9 |
| 11 | F | | 22 | pentalogy of Fallot | ND | ND | 22 | ND | Single PGL | 10 |
| 12 | M | | 18 | SV, complete ECD, secundum atrial septal defect, TGA, PS, dextrocardia, bilateral SVC | ND | ND | 18 | ND | Single PGL | 11 |
| 13 | F | | 20 | TOF | ND | 75 | 20 | ND | Single PGL | 12 |
| 14 | F | | 32 | DORV, VSD, PAS | 80 | ND | 32 | ND | Single PGL | 13 |
| 15 | F | | 41 | SV, TGA, PAH | 82 | ND | 41 | NE | Single PGL | 14,15 |
| 16 | F | | 32 | TOF | 85 | 56 | 32 | ND | Single PGL | 16 |
| **Cont.** |  | |  |  |  |  |  |  |  |  |
| 17 | M | | 22 | TGA | ND | ND | 22 | ND | Single PGL with metastases | 17 |
| 18 | F | | 23 | dextrocardia, SV, PA | 87.4 | 48.8 | 23 | NE | Single PGL with metastases | 18 |
| 19 | F | | 43 | TA, TGA | 80 | 52 | 43 | NE | Single PGL with metastases | 19 |
| 20 | M | | 16 | DORV, PS | 43 | ND | 16 | ND | Single PHEO | 7 |
| 21 | M | | 44 | Ebstein's anomaly of tricuspid valve | 90 | ND | 44 | NE | Single PHEO | 7 |
| 22 | M | | 16 | TA, pulmonary vascular stenosis, VSD, ASD | 80 | 42.6 | 16 | NE | Single PHEO | 20 |
| 23 | F | | 45 | TOF | 94 | ND | 45 | NE | Single PHEO | 21 |
| 24 | F | | 41 | TOF | 72 | ND | 41 | NE | Single PHEO | 21 |
| 25 | F | | 27 | DORV, PA, hypoplastic left ventricle | 78-83 | ND | 27 | NE/DA | Single PHEO | 22 |
| 26 | M | | 20 | double-inlet single left ventricle | ND | ND | 20 | NE/DA | Single PHEO | 23 |
| 27 | M | | 21 | DORV | 74 | ND | 21 | NE | Single PHEO | 24 |
| 28 | M | | 19 | SV, SA | 95 | ND | 19 | NE/DA | Single PHEO | 24 |
| 29 | M | | 13 | SV, SA, bilateral SVC, PS, PDA | 78 | ND | 13 | NE | Single PHEO | 5 |
| 30 | F | | 14 | SV with double inlet, PA | ND | 44 | 14 | NE | Single PHEO | 25 |
| 31 | F | | 29 | TOF | 68 | ND | 29 | ND | Single PHEO | 26 |
| 32 | F | | 33 | TOF | 84 | ND | 33 | NE | Single PHEO | 27 |
| 33 | F | | 34 | TOF, PDA | 72 | ND | 34 | NE | Single PHEO | 28 |
| 34 | F | | 14 | TOF | ND | ND | 14 | ND | Single PHEO | 29 |
| 35 | F | | 25 | TOF | 70 | 24 | 25 | NE/E | Single PHEO | 30 |
| 36 | M | | 29 | situsinversus, TOF | 92 | ND | 29 | E | Single PHEO | 31 |
| 37 | F | | 15 | type Ic TA | 80 | ND | 15 | NE | Single PHEO | 32 |
|  |  | |  |  |  |  |  |  |  |  |
| **Cont.** | |  |  |  |  |  |  |  |  |  |
| 38 | F | | 14 | TGA | 77 | ND | 14 | ND | Single PHEO with metastases | 7 |
| 39 | F | | 28 | Hypoplasia of the tricusoid and pulmonary valves, hypoplastic right ventricle, ASD | 74 | ND | 28 | ND | Single PGL | 33 |
| 40 | F | | 28 | complete AV canal defect, common AV valve, bilateral SVC, common atrium | 72 | 37 | 28 | NE/E/DA | Single PHEO | 34 |
| 41 | F | | 35 | ASD, PDA, PAH | ND | ND | 32 | NE | Single PGL | 35 |
| 42 | F | | 36 | Eisenmenger's syndrome | ND | ND | 16 | ND | Single PGL | 36 |
| 43 | F | | 34 | Eisenmenger's syndrome | 80 | ND | ND | ND | Multiple PHEO | 37 |
| 44 | F | | 42 | Eisenmenger's syndrome | 85 | ND | ND | ND | Single PGL | 38 |
| 45 | F | | 58 | Eisenmenger's syndrome | 80 | 54 | ND | ND | Single PGL | 39 |
| 46 | F | | 59 | Eisenmenger's syndrome | ND | ND | ND | ND | Single PGL | 40 |
| 47 | F | | 60 | Eisenmenger's syndrome | 90 | ND | ND | ND | Single PGL | 41 |

Abbreviations: SV, single ventricle; TA, tricuspid atresia; PS, pulmonary stenosis; SVC, superior vena cava; DORV, double-outlet right ventricle; VSD, ventricular septal defect; TGA, transposition of the great arteries; IVC, inferior vena cava; SA, single atrium; PA, pulmonary atresia; ASD, atrial septal defect; TOF, tetralogy of Fallot; ECD, endocardial cushion defect; PAS, pulmonary artery stenosis; PAH, pulmonary arterial hypertension; PDA, patent ductus arteriosus; ND, no data; NE, norepinephrine; E, epinephrine; DA, dopamine; PHEO, pheochromocytoma; PGL, paraganglioma.

**References**

1. Hirsch JH, Killien FC, Troupin RH. Bilateral carotid body tumors and cyanotic heart disease*. *AJR Am J Roentgenol.* 1980;134(5):1073-1075.

2. Cherqaoui I, Raux O, Dehour L, Rochette A, Dadure C, Capdevila X. Transpulmonary thermodilution hemodynamic monitoring for pheochromocytoma surgery in a child with complex congenital heart disease. *Paediatr Anaesth.* 2006;16(12):1277-1280.

3. Das S, Kumar S, Nath M, Bhalla AP. Perioperative management of combined surgery for phaeochromocytoma and double outlet right ventricle: A rare combination. *Indian J Anaesth.* 2015;59(6):378-380.

4. Reynolds JL, Gilchrist TF. Congenital heart disease and pheochromocytoma. *American journal of diseases of children (1960).* 1966;112(3):251-255.

5. Chung SJ LA, Shin CH, Yang SW, Bae EJ, Noh JII. {Pheochromocytoma associated with cyanotic congenital heart disease}. *Korean J Pediatr.* 2008.

6. Filgueiras-Rama D, Oliver JM, Ruiz-Cantador J, et al. Pheochromocytoma in Eisenmenger's syndrome: a therapeutic challenge. *Rev Port Cardiol.* 2010;29(12):1873-1877.

7. Folger GM, Jr., Roberts WC, Mehrizi A, et al. Cyanotic Malformations of the Heart with Pheochromocytoma. A Report of Five Cases. *Circulation.* 1964;29:750-757.

8. Wilmshurst P, Newbegin C, Paes R. Chemodectoma in a patient with a single ventricle. *Heart (British Cardiac Society).* 1997;77(4):385.

9. Zhang F, Gu W, Dong R, Dong Q, Yu B. Anesthetic management of pheochromocytoma resection in a patient with F4 and a complete endocardial cushion defect. *Acta Anaesthesiol Scand.* 2009;54(6):785-786.

10. Gabhane SK, Gangane NM, Sinha RT. Pentalogy of Fallot and cardiac paraganglioma: a case report*. *Cases journal.* 2009;2:9392.

11. Hwang BH, Kim HY, Jung SE, Park KW. Extra-adrenal pheochromocytoma after operation of congenital heart disease: a case report of 18-year-old boy. *J Korean Surg Soc.* 2012;83(1):65-68.

12. Yildiz BS, Sasmazel A, Baysal A, et al. Assessment of carotid body tumor and its association with tetralogy of fallot: effect of the chronic hypoxia. *Heart views : the official journal of the Gulf Heart Association.* 2014;15(3):86-88.

13. Kohno M, Nagamine Y, Goto T. [A Case of Undiagnosed Extra-adrenal Pheochromocytoma in an Adult Patient with Single Ventricle Circulation after the Bidirectional Glenn Operation]. *Masui.* 2015;64(9):985-988.

14. Oleaga-Alday A, Goni-Goicoechea F, Calles-Romero L, Perez de Ciriza-Cordeu M, Paja-Fano M. Paraganglioma and cyanotic congenital heart disease: The role of tisular hipoxia. *Endocrinologia y nutricion : organo de la Sociedad Espanola de Endocrinologia y Nutricion.* 2015;62(8):413-414.

15. Una Orejon R, Altit Millan E, Aguar Fernandez M, Ureta Tolsada MP. [Catecholamine-secreting paraganglioma in a patient with Eisenmenger syndrome and a single ventricle. Nitric oxide administration and minimally invasive haemodynamic monitoring]. *Revista espanola de anestesiologia y reanimacion.* 2015;63(3):172-176.

16. Patkar CS, Baldwa N, Dave S, Gujjar P. Perioperative anaesthetic management of phaeochromocytoma associated with uncorrected tetralogy of Fallot. *Indian J Anaesth.* 2015;59(12):816-818.

17. Bockelman HW, Arya S, Gilbert EF. Cyanotic congenital heart disease with malignant paraganglioma*. *Cancer.* 1982;50(11):2513-2517.

18. Yoshihara A, Tanabe A, Saito H, et al. A case of malignant pheochromocytoma with Holt-Oram syndrome. *Endocr J.* 2008;55(1):153-159.

19. Subedi R, Judson MA. Chronic hypoxemia and paraganglioma: a rare case and discussion of potential mechanisms. *The American journal of the medical sciences.* 2014;348(6):528-529.

20. Ueda N, Kitamura Y, Hayashi Y, et al. Anaesthetic management of phaeochromocytoma associated with tricuspid atresia. *Can J Anaesth.* 1991;38(6):780-784.

21. Kita T, Imamura T, Date H, et al. Two cases of pheochromocytoma associated with tetralogy of Fallot. *Hypertens Res.* 2003;26(5):433-437.

22. Sparks JW, Seefelder C, Shamberger RC, McGowan FX. The perioperative management of a patient with complex single ventricle physiology and pheochromocytoma. *Anesth Analg.* 2005;100(4):972-975.

23. Yuki K, Shamberger RC, McGowan FX, Jr., Seefelder C. The perioperative management of a patient with Fontan physiology for pheochromocytoma resection. *J Cardiothorac Vasc Anesth.* 2007;22(5):748-750.

24. Abe I, Nomura M, Sakamoto R, et al. [Two cases of pheochromocytoma associated with single ventricle syndrome]. *Nihon Naika Gakkai zasshi The Journal of the Japanese Society of Internal Medicine.* 2007;96(5):994-996.

25. Cheung YW, Spevack DM. Single left ventricle and pheochromocytoma. *Congenit Heart Dis.* 2008;3(5):355-358.

26. Balakrishnan G, Ravikumar R, Rao S, Balakrishnan KR. Tetralogy of Fallot with pheochromocytoma: an unusual therapeutic challenge. *Asian Cardiovasc Thorac Ann.* 2013;21(4):464-466.

27. Rich BS, Moo TA, Mark S, et al. Sympathetic paraganglioma in a patient with unrepaired tetralogy of Fallot: a case report and review of the literature. *J Clin Endocrinol Metab.* 2013;98(1):7-12.

28. Tang YK, Flora Tsang HF, Ranjan Das S, Vance ML, Kussman BD. Case 6-2013: Perioperative management of an adult patient with tetralogy of fallot and pheochromocytoma. *J Cardiothorac Vasc Anesth.* 2013;27(6):1399-1406.

29. Kasaliwal R, Sarathi V, Pandit R, et al. Pheochromocytoma and tetralogy of Fallot: a rare but potentially dangerous combination*. *Endocr Pract.* 2014;20(5):e80-85.

30. Singh GD, Anuradha S, Sethi P, Singla S, Saran RK, Dewan R. Pheochromocytoma and tetralogy of Fallot: Coincidence or a rare association? *Asian Cardiovasc Thorac Ann.* 2014;24(1):75-77.

31. Tapia-Orihuela RK, Huaringa-Marcelo J, Loja-Oropeza D. Tetralogy of Fallot and pheochromocytoma in a situs inversus totalis: An unusual association. *J Cardiovasc Thorac Res.* 2016;8(3):132-136.

32. Yamamoto K, Namba N, Kubota T, et al. Pheochromocytoma complicated by cyanotic congenital heart disease: a case report. *Clin Pediatr Endocrinol.* 2016;25(2):59-65.

33. Nissenblatt MJ. {Cyanotic heart disease: "low altitude" risk for carotid body tumor?}. *The Johns Hopkins medical journal.* 1978;142(1):18-22.

34. Bellingham GA, Dhir AK, Luke PP. Case report: retroperitoneoscopic pheochromocytoma removal in an adult with Eisenmenger's syndrome. *Can J Anaesth.* 2008;55(5):295-301.

35. Xi JB，Tian XQ. Case report: congenital heart disease with pheochromocytoma. *Clinical Misdiagnosis and Mistherapy.* 2012;25(2):91-92.

36. Kraayenbrink MA, Steven CM. Anaesthesia for carotid body tumour resection in a patient with the Eisenmenger syndrome. A case report. *Anaesthesia.* 1985;40(12):1194-1197.

37. Rutter TW, Mullin V. Pheochromocytoma in a patient with Eisenmenger's complex. *Anesth Analg.* 1991;73(4):496-498.

38. Jones HG, Stoneham MD. Continuous cervical plexus block for carotid body tumour excision in a patient with Eisenmenger's syndrome. *Anaesthesia.* 2006;61(12):1214-1218.

39. Bhagwan S, Cortes M, Gooden C, Neustein SM. Regional anesthesia for carotid surgery in an adult patient with cyanotic congenital heart disease. *J Cardiothorac Vasc Anesth.* 2009;24(2):312-313.

40. Hsu CC, Singh D, Kwan GN, Bhuta S. 18F-FDG PET/CT in a Patient With Glomus Vagale Paraganglioma and Eisenmenger Syndrome: Searching for the Missing Link? *Clinical nuclear medicine.* 2016;41(3):e135-136.

41. Mak JK, Kay M. Carotid body tumour associated with cyanotic heart disease. *BMJ case reports.* 2016;2016.
